# Post–COVID-19 Condition in Children 6 and 12 Months After Infection

**DOI:** 10.1001/jamanetworkopen.2023.49613

**Published:** 2023-12-28

**Authors:** Frederick Dun-Dery, Jianling Xie, Kathleen Winston, Brett Burstein, Jocelyn Gravel, Jason Emsley, Vikram Sabhaney, Roger Zemek, Simon Berthelot, Darcy Beer, April Kam, Gabrielle Freire, Ahmed Mater, Robert Porter, Naveen Poonai, Anne Moffatt, Andrew Dixon, Marina I. Salvadori, Stephen B. Freedman

**Affiliations:** 1 Department of Pediatrics, Cumming School of Medicine, University of Calgary, Calgary, Alberta, Canada; 2 Section of Pediatric Emergency Medicine, Department of Pediatrics, Cumming School of Medicine, University of Calgary, Calgary, Alberta, Canada; 3 Department of Pediatrics, Cumming School of Medicine, University of Calgary, Calgary, Alberta, Canada; 4 Division of Pediatric Emergency Medicine, Department of Pediatrics, Montreal Children’s Hospital, McGill University Health Centre, Montreal, Quebec, Canada; 5 Department of Epidemiology, Biostatistics, and Occupational Health, McGill University, Montreal, Quebec, Canada.; 6 Department of Pediatric Emergency Medicine, Centre Hospitalier Universitaire (CHU) Sainte-Justine, Université de Montréal, Montreal, Quebec, Canada; 7 Department of Emergency Medicine, IWK Children’s Health Centre and Queen Elizabeth II Health Sciences Centre, Dalhousie University, Halifax, Nova Scotia, Canada; 8 Departments of Paediatrics and Emergency Medicine, BC Children’s Hospital, University of British Columbia, Vancouver, British Columbia, Canada; 9 Department of Pediatrics, University of Ottawa, Children’s Hospital of Eastern Ontario, Ottawa, Ontario, Canada; 10 Department of Emergency Medicine, University of Ottawa, Children’s Hospital of Eastern Ontario, Ottawa, Ontario, Canada; 11 Département de médecine de famille et de médecine d’urgence, CHU de Québec-Université Laval, Québec City, Quebec, Canada; 12 Department of Pediatrics and Child Health, The Children’s Hospital of Winnipeg, Children’s Hospital Research Institute of Manitoba, University of Manitoba, Winnipeg, Canada; 13 Division of Emergency Medicine, Department of Pediatrics, McMaster Children’s Hospital, Hamilton, Ontario, Canada; 14 Division of Emergency Medicine, Department of Paediatrics, Hospital for Sick Children, Faculty of Medicine, University of Toronto, Toronto, Ontario, Canada; 15 Section of Pediatric Emergency, Department of Pediatrics, Jim Pattison Children’s Hospital, University of Saskatchewan, Saskatoon, Saskatchewan, Canada; 16 Janeway Children’s Health and Rehabilitation Centre, Newfoundland and Labrador Health Services, St John’s, Newfoundland and Labrador, Canada; 17 Department of Paediatrics, Children’s Hospital London Health Sciences Centre, Schulich School of Medicine and Dentistry, London, Ontario, Canada; 18 Department of Internal Medicine, Schulich School of Medicine and Dentistry, London, Ontario, Canada; 19 Department of Epidemiology and Biostatistics, Schulich School of Medicine and Dentistry, London, Ontario, Canada; 20 Department of Paediatrics, Kingston Health Sciences Centre, Queen’s University, Kingston, Ontario, Canada; 21 Section of Pediatric Emergency Medicine, Departments of Pediatric, Women’s and Children’s Health Research Institute, University of Alberta, Edmonton, Canada; 22 Public Health Agency of Canada, Ottawa, Ontario, Canada; 23 Department of Pediatrics, McGill University, Montreal, Quebec, Canada; 24 Sections of Pediatric Emergency Medicine and Gastroenterology, Departments of Pediatrics and Emergency Medicine, Cumming School of Medicine, University of Calgary, Calgary, Alberta, Canada

## Abstract

**Importance:**

There is a need to understand the long-term outcomes among children infected with SARS-CoV-2.

**Objective:**

To quantify the prevalence of post–COVID-19 condition (PCC) among children tested for SARS-CoV-2 infection in pediatric emergency departments (EDs).

**Design, Setting, and Participants:**

Multicenter, prospective cohort study at 14 Canadian tertiary pediatric EDs that are members of the Pediatric Emergency Research Canada network with 90-day, 6-month, and 12-month follow-up. Participants were children younger than 18 years who were tested for SARS-CoV-2 infection between August 2020 and February 2022. Data were analyzed from May to November 2023.

**Exposure:**

The presence of SARS-CoV-2 infection at or within 14 days of the index ED visit.

**Main Outcomes and Measures:**

Presence of symptoms and QoL reductions that meet the PCC definition. This includes any symptom with onset within 3 months of infection that is ongoing at the time of follow-up and affects everyday functioning. The outcome was quantified at 6 and 12 months following the index ED visit.

**Results:**

Among the 5147 children at 6 months (1152 with SARS-CoV-2 positive tests and 3995 with negative tests) and 5563 children at 12 months (1192 with SARS-CoV-2 positive tests and 4371 with negative tests) who had sufficient data regarding the primary outcome to enable PCC classification, the median (IQR) age was 2.0 (0.9-5.0) years, and 2956 of 5563 (53.1%) were male. At 6-month follow-up, symptoms and QoL changes consistent with the PCC definition were present in 6 of 1152 children with positive SARS-CoV-2 tests (0.52%) and 4 of 3995 children with negative SARS-CoV-2 tests (0.10%; absolute risk difference, 0.42%; 95% CI, 0.02% to 0.94%). The PCC definition was met at 12 months by 8 of 1192 children with positive SARS-CoV-2 tests (0.67%) and 7 of 4371 children with negative SARS-CoV-2 tests (0.16%; absolute risk difference, 0.51%; 95% CI, 0.06 to 1.08%). At 12 months, the median (IQR) PedsQL Generic Core Scale scores were 98.4 (90.0-100) among children with positive SARS-CoV-2 tests and 98.8 (91.7-100) among children with negative SARS-CoV-2 tests (difference, −0.3; 95% CI, −1.5 to 0.8; *P* = .56). Among the 8 children with SARS-CoV-2 positive tests and PCC at 12-month follow-up, children reported respiratory (7 of 8 patients [88%]), systemic (3 of 8 patients [38%]), and neurologic (1 of 8 patients [13%]) symptoms.

**Conclusions and Relevance:**

In this cohort study of children tested for SARS-CoV-2 infection in Canadian pediatric EDs, although children infected with SARS-CoV-2 reported increased chronic symptoms, few of these children developed PCC, and overall QoL did not differ from children with negative SARS-CoV-2 tests.

## Key Points

**Question:**

What proportion of children meet the post–COVID-19 condition (PCC) symptom definition at 6 and 12 months following SARS-CoV-2 testing in pediatric emergency departments?

**Findings:**

In this cohort study, at 6 and 12 months, a statistically greater number of children with SARS-CoV-2 positive tests compared with those with negative tests met the PCC symptom and quality of life change definition. However, the absolute risk differences were very small (0.42% and 0.51% at 6 and 12 months, respectively).

**Meaning:**

Although there is an increased prevalence of symptoms consistent with the PCC definition that reduce quality of life among SARS-CoV-2 infected children, very few infected children develop PCC.

## Teaser

This cohort study evaluates the prevalence of post–COVID-19 condition among children tested for SARS-CoV-2 infection in pediatric emergency departments in Canada.

## Introduction

Although post–COVID-19 condition (PCC) is a recognized entity, an accurate estimate of how frequently it develops and persists remains unclear, particularly among children. The most commonly used broad, nonspecific definition (new or persistent symptom[s] lasting 2 or more months, beginning within 3 months of confirmed or probable SARS-CoV-2 infection not better explained by another health condition^1^) has led to widely differing prevalence estimates among adults (range: 7.5%^2^ to 45%^3,4^). A systematic review^5^ suggests the risk may be as high as 25% in children. These differing estimates and the conclusions of meta-analyses are limited due to heterogeneity in populations, designs, data collection methods, and definitions.

Although prospective pediatric studies that include control participants report lower PCC rates, lower symptom duration in children than among adults,^6^ and higher PCC prevalence in adolescents compared with younger children,^7^ these findings are inconsistent. While a study of 2368 children with SARS-CoV-2 seeking emergency department (ED) care reported an absolute increased risk of 1.6% for PCC at 90 days compared with matched controls,^8^ in another study, 25% of adolescents with positive tests and 18% of those with negative tests reported PCC symptoms 6 months after infection.^9^ In a German study,^10^ the prevalence of moderate or severe postinfection symptoms among adolescent girls was 32% among individuals with infection compared with 9% among those without at 12-month follow-up.

To address the aforementioned limitations and to standardize pediatric PCC definitions, the World Health Organization (WHO) adopted a consensus definition that added several qualifiers, notably, that symptoms have an onset within 3 months of the infection, persist for a minimum of 2 months, and limit everyday function and ascertainment of developmental milestones.^11,12^ Use of this definition has the potential to advance our understanding of PCC in children. Thus, we sought to quantify the prevalence of PCC at 6 and 12 months after acute infection and to characterize symptoms among children evaluated in pediatric EDs across Canada using the WHO definition.

## Methods

### Study Design and Setting

This prospective cohort study recruited participants between August 4, 2020, and February 22, 2022, in 14 Pediatric Emergency Research Canada tertiary-care pediatric EDs.^13^ Participating institutions (eTable 1 in Supplement 1) obtained research ethics board approval; informed oral consent was obtained and participant permission was required according to institutional policy. Study details are reported in accordance with the Strengthening the Reporting of Observational Studies in Epidemiology (STROBE) reporting guidelines.^14^

### Participants and Recruitment

Children younger than 18 years who underwent testing for SARS-CoV-2 because of symptoms or epidemiologic risk factors were eligible. Specimens were collected at the discretion of the treating physician and were analyzed per local laboratory standards.

Research team members received a daily list of potentially eligible children. Research assistants consecutively attempted to contact all children who tested positive for SARS-CoV-2 by telephone, followed by consecutive phone calls to children who tested negative. They started with the first child tested each day in each of the categories to minimize selection bias. On certain days, the number of potentially eligible participants exceeded research team member capacity and thus not all potential participants could be approached.

### Outcomes

#### Primary

The primary outcome was the proportion of SARS-CoV-2–positive participants with PCC at 6 and 12 months following SARS-CoV-2 testing.^12^ Our PCC definition was operationalized by aligning the WHO PCC definition^11,12^ with our database by requiring that (1) caregivers reported the presence of a chronic sign, symptom, or diagnosis within the preceding 3 months that manifested within 2 months of the 90-day follow-up survey; (2) overall health status (0- to 100-point scale) reported on the 12-month survey was lower than before the index ED visit; and (3) for children aged 2 years or older, everyday functioning had to be reduced as quantified on a quality of life (QoL) score (Table 1).

**Table 1.** Operational Definition of Post–COVID-19 Condition Compared With the World Health Organization in Children and Adolescents

Criteria	World Health Organization definition^1^	Study definition
Population	Children and adolescents	<18 y
Evidence of COVID-19 infection	History of probable or confirmed SARS-CoV-2 infected persons	Polymerase chain reaction positive nares, nasopharyngeal, or oral swab specimen collected at the index emergency department visit or within the subsequent 14 d
Duration of symptoms	Lasting ≥2 mo	Reported 9 to 13 mos after the index illness^a^
Symptom onset	Within 3 mo of probable or confirmed SARS-CoV-2 infection	Reported 30 to 90 d after the index illness
Severity	Have a change in everyday function and developmental milestones	Overall health status at 12-mo follow-up as rated on a 0- to 100-point scale is lower than it was before the index illness, and the total PedsQL score must be categorized as suboptimal (<78.6)^21^ on the average score across the psychosocial and physical functioning domains^b^

Abbreviation: PedsQL, Pediatric Quality of Life Inventory Generic Core Scale.

^a^ For 6-month analysis, the time used was 3 to 7 months after the index emergency department visit.

^b^ The PedsQL score was calculated using data reported at the 6-month follow-up survey. Children less than 2 years were not required to meet the PedsQL criteria as the tool is not validated for use in that population.^19^

#### Secondary

The first secondary outcome was QoL, measured using the Pediatric Quality of Life Inventory-Version 4.0 (PedsQL-4.0) survey tool.^15^ The second was QoL as reported on a 0- to 100-point scale, and the third was symptom profiles of children with PCC at 12 months.

### Data Collection

Demographic and medical information was collected via caregiver interviews following the index ED visit and 14 days following the ED visit. At the 90-day interview, information was collected regarding any chronic symptoms, signs, and/or diagnoses Medical record review was performed to confirm SARS-CoV-2 test result status, index ED visit disposition, and 14-day outcome data. Six and 12 months after the index ED visit, caregivers were administered a modified version of the International Severe Acute Respiratory and emerging Infection Consortium Long-COVID Pediatric Questionnaire (eTable 2 in Supplement 1).^16^ As the importance of PCC was underappreciated at the time of study launch, 6- and 12-month follow-up surveys were added starting on November 1, 2021. Surveys could be completed up to a maximum of 30 days following the 6- and 12-month time points.

### Definitions

SARS-CoV-2 status was classified as positive if a nucleic acid test performed on a swab obtained from the nares, nasopharynx, or oral cavity at the index ED visit or during the subsequent 14 days was positive. Participants with negative tests constituted the comparison group. Acute SARS-CoV-2 hospitalization status incorporated events occurring until 14 days after the index ED visit.^17^ Testing and reporting of variant of concern (VOC) varied by institution and over time. When a VOC or a variant linked to a VOC was identified, that report was used for classification purposes. If VOC testing was not performed or results were inconclusive, the SARS-CoV-2 variant was classified as previously described.^18^

We assessed QoL using age-specific PedsQL-4.0 Generic Core Scales^19,20^ which include 4 subscales focused on physical, emotional, social, and school functioning.^21^ Each question uses a 5-point Likert response scale ranging from never to almost always for participants to report how much of a problem their child had during the preceding 7 days. In accordance with recommended approaches,^20,22^ individual item scores were transformed to corresponding values (never = 100, almost never = 75, sometimes = 50, often = 25, almost always = 0), with higher scores indicating better health-related QoL. Domain scores were obtained by adding the sum of items and then dividing by the number of items answered.^19,20^ Total PedsQL scores were calculated by taking the mean of the 4 subdomains.^15,19^

An individual’s condition was deemed to have reduced their daily functioning if their total PedsQL score was greater than 1 minimum clinically important difference (MCID) below the mean total score of healthy children.^15^ The PedsQL MCID (4.4 points) is the smallest difference that patients perceive to be relevant, and would mandate a change in care.^15^ Because the mean score of healthy children is 83 points,^15,21^ a PedsQL total score below 78.6 was defined as suboptimal or abnormal and consistent with the WHO PCC criteria of impaired daily functioning.^12^ For children younger than 2 years, QoL assessment was limited to caregiver rating on the 0- to 100-point scale.

### Sample Size

According to prior work in a similar population,^8^ we estimated that 5% of SARS-CoV-2–positive participants would experience PCC. Thus, 812 participants with positive SARS-CoV-2 tests were required to estimate the true PCC prevalence with 95% confidence within ±1.5% of the measured value, and 1092 participants in each group were required to provide 80% power to detect a 2.5% between-group PCC difference with 95% confidence.

### Statistical Analyses

Data were summarized using descriptive statistics, and baseline categorical variables were compared using the χ^2^ test or the Fisher exact test as appropriate; the Mann-Whitney *U* test was used for continuous variables as they were not normally distributed. For the outcome of PCC at 6 and 12 months, we compared event rates using the Fisher exact test. The Wald test was used to obtain the 95% CI of the difference between proportions, and the Agresti-Caffo approach^23^ was used when the event was rare (<10 participants). As the PCC definition requires symptom onset within 90 days, along with symptoms at the follow-up time point, participants who reported the absence of symptoms on the 90-day survey were classified as PCC negative at 6- and 12-month follow-up, regardless of symptoms reported on the latter 2 surveys. Similarly, those who reported the absence of symptoms at 6- and 12-month follow-up were classified as PCC negative regardless of symptoms reported at 90-day follow-up. Three sensitivity analyses of the primary outcome were performed. The first used a revised definition of PCC that did not require that symptoms initially manifest within 30 to 90 days of the index visit. The second excluded children who were asymptomatic at the 90-day survey but failed to complete the 12-month survey. The third removed the requirement of a lower overall health status score at the 12-month follow-up survey relative to the reported overall health status preceding the index illness.

QoL measured using the PedsQL and 100-point scale were compared between groups using the Mann-Whitney *U* test, and the CIs of the difference were calculated using median regression. We compared the proportions of children who rated their overall health status as lower at follow-up than preceding their index visit and those who had PedsQL scores less than 78.6 using Fisher Exact tests. Symptom profiles of children with PCC at 12 months are reported descriptively.

When assessing the presence or absence of individual baseline symptoms, those with missing values were considered not present.^24^ Missing data values for key variables are reported in eTable 3 in Supplement 1. All analyses were 2-sided, and statistical significance was defined by a *P *value less than .05. *P* values obtained from unadjusted bivariate analyses of the primary and secondary outcomes were adjusted for multiple comparisons via the Benjamini-Hochberg approach.^25^ Analyses were performed using SPSS Statistics for Windows, version 25 (IBM Corporation) and Stata version 17.0 (StataCorp). Data were analyzed from May to November 2023.

## Results

### Participant Characteristics

The 12-month follow-up cohort included 1192 of 1454 potentially eligible participants with SARS-CoV-2 positive tests (82.0%) and 4371 of 5809 participants with negative tests (75.3%) (Figure). Median (IQR) age of study participants was 2.0 (0.9-5.0) years, 2956 of 5563 (53.1%) were male, and 170 of 2917 (5.8%) had received 1 or more COVID-19 vaccine doses (Table 2; eTable 4 in Supplement 1). Those lost to follow-up were more likely infected by the wild-type strain and less likely to have Omicron (eTable 5 and eTable 6 in Supplement 1).

**Figure.**
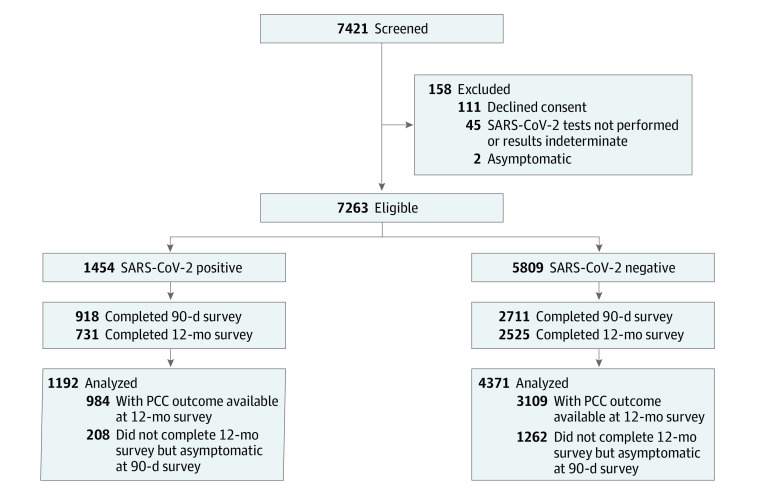
Flow Diagram of Participants From Index Emergency Department Enrollment Visit to 12-Month Follow-Up PCC indicates post–COVID-19 condition; among SARS-CoV-2 negative participants, use of this term refers to the presence of symptoms consistent with PCC.

**Table 2.** Baseline Comparisons of Participants Who Completed the 12-Month Follow-Up Survey, Stratified by Index Emergency Department Visit SARS-CoV-2 Status

Characteristics	Participants, No./total No. (%)		
	All (N = 5563)	SARS-CoV-2 positive (n = 1192)	SARS-CoV-2 negative (n = 4371)
Age, median (IQR), y	2.0 (0.9-5.0)	2.0 (0.5-7.0)	2.0 (1.0-5.0)
Age			
<2 y	2612/5563 (47.0)	592/1192 (49.7)	2020/4371 (46.2)
2 to <5 y	1407/5563 (25.3)	214/1192 (18.0)	1193/4371 (27.3)
5 to <8 y	578/5563 (10.4)	123/1192 (10.3)	455/4371 (10.4)
8 to <13 y	592/5563 (10.6)	171/1192 (14.3)	421/4371 (9.6)
13 to 18 y	374/5563 (6.7)	92/1192 (7.7)	282/4371 (6.5)
Sex			
Female	2607/5563 (46.9)	531/1192 (44.6)	2076/4371 (47.5)
Male	2956/5563 (53.1)	661/1192 (55.5)	2295/4371 (52.5)
Chronic underlying condition (excluding asthma)	722/5558 (13.0)	181/1189 (15.2)	541/4369 (12.4)
History of asthma	522/5559 (9.4)	83/1189 (7.0)	439/4370 (10.1)
COVID-19 vaccination received before index visit^a^			
No	2570/2917 (88.1)	691/839 (82.4)	1879/2078 (90.4)
≥1 Dose of any approved vaccine	170/2917 (5.8)	69/839 (8.2)	101/2078 (4.9)
Unknown	177/2917 (6.1)	79/839 (9.4)	98/2078 (4.7)
Variant of concern			
Wild type	NA	322/1192 (27.0)	NA
Alpha	NA	189/1192 (15.9)	NA
Gamma	NA	4/1192 (0.34)	NA
Delta	NA	259/1192 (21.7)	NA
Omicron	NA	418/1192 (35.1)	NA
Hospitalized for the acute illness^b^	543/5563 (9.8)	136/1192 (11.4)	407/4371 (9.3)
ICU admission during the acute illness^b^	36/5563 (0.65)	8/1192 (0.67)	28/4371 (0.64)
Antibiotics during the acute illness^b^	1008/5542 (18.2)	172/1187 (14.5)	836/4355 (19.2)
Corticosteroids during the acute illness^b^	853/5539 (15.4)	150/1182 (12.7)	703/4357 (16.1)

Abbreviations: ICU, intensive care unit; NA, not applicable.

^a^ The question was implemented to the study on June 11, 2021, about 10 months after the study was officially launched.

^b^ Incident occurred at or within 14 days of the index emergency department visit.

### Outcomes

At 6-month follow-up, the PCC symptom and QoL definition was met by 6 of 1152 children with positive tests (0.52%; 95% CI, 0.24%-1.13%) and 4 of 3995 children with negative tests (0.10%; 95% CI, 0.04%-0.26%; difference, 0.42%; 95% CI, 0.02%-0.94%). At 12-month follow-up, the primary outcome PCC definition was met by 8 of 1192 children with positive tests (0.67%; 95% CI, 0.34%-1.32%) and 7 of 4371 children with negative tests (0.16%; 95% CI, 0.08%-0.33%; difference, 0.51%; 95% CI, 0.06%-1.08%) (Table 3; eTable 7 in Supplement 1). All 3 sensitivity analyses were consistent with the findings of our primary analysis (eTable 8 in Supplement 1).

**Table 3.** Outcomes According to Index Emergency Department SARS-CoV-2 Test Result Status^a^

Elements defining the composite PCC outcome measure	Participants, No./total No. (%)		Adjusted *P* value^b^	Absolute difference (95% CI of difference)
	SARS-CoV-2 positive	SARS-CoV-2 negative		
12-Month Follow-Up Survey Outcome Data				
Met PCC definition^c^	8/1192 (0.67)	7/4371 (0.16)	.02	0.51% (0.06% to 1.08%)
Any chronic signs/symptoms or diagnoses at 30-90 d following index ED visit	81/1089 (7.4)	204/3915 (5.2)	.01	2.2% (0.5% to 3.9%)
Any chronic signs/symptoms or diagnoses at 9-13 mos following index ED visit	71/727 (9.8)	267/2517 (10.6)	.56	−0.84% (−3.3% to 1.6%)
Overall health status at the time of the index ED visit as rated by caregivers on a 0- to 100-point scale at the time of 12-mo follow-up data collection, median (IQR) [No.]	95.0 (80.0 to 100) [720]	89.0 (60.0 to 100) [2495]	<.001	6.0 (3.2 to 8.8)
Overall health status at the time of 12-mo follow-up as rated by caregivers on a 0- to 100-point scale at the time of 12-mo follow-up data collection, median (IQR) [No.]	95.0 (85.0 to 100) [723]	95.0 (85.0 to 100) [2504]	.45	0 (−2.1 to 2.1)
Overall health status at 12 mo was rated as lower than before the index illness	170/720 (23.6)	374/2495 (15.0)	<.001	8.6% (5.2% to 12.0%)
PedsQL score (age >2 y), median (IQR) [No.]^d^	98.4 (90.0 to 100) [456]	98.8 (91.7 to 100) [1951]	.56	−0.3 (−1.5 to 0.8)
PedsQL score (age >2 y) <78.6^d^	51/456 (11.2)	159/1951 (8.1)	.07	3.0% (−0.1% to 6.2%)
6-Month Follow-Up Survey Outcome Data				
Met PCC definition^c^	6/1152 (0.52)	4/3995 (0.10)	.02	0.42% (0.02% to 0.94%)
Any chronic signs/symptoms or diagnoses at 30-90 d following index ED visit	67/1075 (6.2)	97/3808 (2.5)	<.001	3.7% (2.1% to 5.2%)
Any chronic signs/symptoms or diagnoses at 3-6 mos following index ED visit	35/506 (6.9)	109/1104 (9.9)	.09	−3.0% (−5.8% to −0.1%)
Overall health status at the time of the index ED visit as rated by caregivers on a 0- to 100-point scale at the time of 6-mo follow-up data collection, median (IQR) [No.]	90.0 (60.0 to 100) [501]	90.0 (60.0 to 100) [1092]	.003	0 (−3.0 to 3.0)
Overall health status at the time of 6-mo follow-up as rated by caregivers on a 0- to 100-point scale at the time of 6-mo follow-up data collection, median (IQR) [No.]	99.0 (90.0 to 100) [502]	95.0 (86.8 to 100) [1098]	.006	4.0 (2.4 to 5.6)
Overall health status at 6 mo was rated as lower than before the index illness	84/501 (16.8)	159/1092 (14.6)	.35	2.2% (−1.7% to 6.1%)
PedsQL score (age >2 y), median (IQR) [No.]	100 (91.6 to 100) [282]	100 (93.8 to 100) [706]	.95	0 (−0.9 to 0.9)
PedsQL score (age >2 y) <78.6	25/282 (8.9)	48/706 (6.8)	.35	2.1% (−1.7% to 5.9%)

Abbreviations: ED, emergency department; PedsQL, Pediatric Quality of Life Inventory, Generic Core Scale; PCC, post–COVID-19 condition.

^a^ The presence or absence of PCC could be determined for 1192 children with SARS-CoV-2 positive tests and 4371 children with SARS-CoV-2 negative tests at 12 months (and 1152 and 3995, respectively, at 6 months). As described in the Methods section, not all subelements of the PCC diagnostic criteria are required to permit classification. Thus, although 1192 children with SARS-CoV-2 positive tests and 4371 children with SARS-CoV-2 negative tests had the presence of PCC classified at 12 months, individual element results are reported for varying numbers of participants.

^b^ *P* values were adjusted for multiple comparisons via Benjamini-Hochberg method.

^c^ For participants with SARS-CoV-2 negative tests, use of the PCC term refers to meeting the symptom and quality of life aspects of the definition, but excludes the requirement to test positive for SARS-CoV-2 nucleic acid.

^d^ The reported score was the mean of the total sores of the 4 domains.

At 12-month follow-up, participants with positive SARS-CoV-2 tests reported similar overall health scores (median [IQR] 95 [80-100] before the index ED visit and 95 [85-100] at 12-month follow-up) as reported on a 0- to 100-point scale, while participants with negative tests reported a 6-point median (IQR) increase (from 89 [60-100] to 95 [85-100]). Overall health status at 12 months was rated as lower than before the index ED visit among participants with SARS-CoV-2 positive tests and SARS-CoV-2 negative tests by 23.6% (170 of 720 participants) and 15.0% (374 of 2495 participants), respectively (difference, 8.6%; 95% CI, 5.2% to 12.0%). Median (IQR) PedsQL Generic Core Scale scores were 98.4 (90.0-100) among children with positive SARS-CoV-2 tests and 98.8 (91.7-100) among children with negative SARS-CoV-2 tests (difference, −0.3; 95% CI, −1.5 to 0.8). The proportion of participants older than 2 years with PedsQL scores less than 78.6 was 11.2% (51 of 456 participants) and 8.1% (159 of 1951 participants) among those with positive and negative tests, respectively (difference, 3.0%; 95% CI, −0.1% to 6.2%). Additionally, a greater proportion of children with SARS-CoV-2 positive tests (17% and 24% at 6 and 12 months, respectively) compared with children with negative tests (15% at both time points) reported their child’s overall health was worse on a 0- to 100-point scale, compared with before the index ED visit illness.

Within 7 days of each follow-up time point, 301 of 3241 participants at 12 months (9.3%) compared with 102 of 1502 at 6 months (6.8%) reported being febrile (difference, 2.5%; 95% CI, 0.8%-4.1%) (eTable 9 in Supplement 1). Children with positive tests with PCC at 12-month follow-up reported respiratory (7 of 8 participants [88%]), systemic (3 of 8 participants [38%]), and neurologic (1 of 8 participants [13%]) symptoms (Table 4). The children with negative SARS-CoV-2 tests who met the PCC definition at 12 months reported persistent epilepsy (2 of 7 participants [29%]), systemic concerns (2 of 7 participants [29%]), recurrent fevers (2 of 7 participants [29%]), and the development of asthma (1 of 7 participants [14%]). Symptoms reported by study participants, irrespective of PCC status, according to age and follow-up interval, are reported in eTable 10 in Supplement 1.

**Table 4.** Symptoms at Follow-Up Among Study Participants With Post–COVID-19 Condition at 12 Months^a^

Age at time of index ED visit^b^	Baseline	Day 90			Month 12			
	Any chronic conditions	Respiratory	Neurologic	Other symptoms	Respiratory	Neurologic	Other symptoms	PedsQL score at month 12
SARS-CoV-2 positive								
<3 Mo	No	Recurrent respiratory infections	No	No	Recurrent respiratory infections	No	No	NA
<3 Mo	No	Recurrent respiratory infections	No	No	Recurrent otitis media and pneumonia	No	No	NA
3 Mo to 1 y	No	Nasal congestion	No	No	Adenoidal hypertrophy, otitis media	No	Sleep apnea	76.5
3 Mo to 1 y	No	Recurrent respiratory infections	No	No	Shortness of breath	No	No	NA
1 Y to 5 y^c^	No	Shortness of breath	No	No	No	No	No	58.0
1 Y to 5 y	No	Persistent cough	No	No	Recurrent respiratory infections, chest pain, shortness of breath	No	No	75.8
5 Y to 9 y	Rett syndrome	Recurrent pneumonia	No	No	Recurrent respiratory infections, hypoxia, increased secretions	Increased seizure frequency and intensity	Fatigue	67.9
5 Y to 9 y	No	No	No	Recurrent headache, eye pain, chest pain, and abdominal pain	Cough	No	Recurrent headache and myalgias	78.0
SARS-CoV-2 negative								
3 Mo to 1 y	No	Chronic cough	No	No	Asthma	No	No	NA
3 Mo to 1 y	No	No	No	Dermoid cyst	Asthma, shortness of breath, chronic cough and rhinorrhea.	No	No	NA
3 Mo to 1 y	No	No	No	Recurrent fevers	No	No	Recurrent fevers	NA
1 Y to 5 y	No	No	Epilepsy	No	No	Refractory epilepsy	No	71.2
1 Y to 5 y	No	Chronic cough, recurrent otitis media	No	No	Asthma	No	No	77.4
1 Y to 5 y	Hypothyroidism	Chronic cough, nasal congestion	No	No	Recurrent respiratory infections and pneumonia	No	Anorexia, weight loss, recurrent headaches, and fever	69.5
5 Y to 9 y	Autism, attention deficit hyperactivity disorder, chromosomal abnormality, Tourette syndrome	No	Epilepsy	No	No	Epilepsy	No	15.8

Abbreviations: NA, not applicable; PedsQL, Pediatric Quality of Life Inventory, Generic Core Scale.

^a^ For participants with SARS-CoV-2 negative tests, use of the post–COVID-19 condition term refers to meeting the symptom and quality of life aspects of the definition but excludes the requirement to test positive for SARS-CoV-2 nucleic acid.

^b^ Age groups provided instead of exact ages to preserve anonymity of patients.

^c^ Although the survey respondent indicated the presence of a chronic sign, symptom, or diagnosis (assigned by a medical professional) that may have been associated with the acute COVID-19 infection 9 to 13 months after the index illness, the respondent did not specify which sign, symptom, or diagnosis was present.

## Discussion

In this prospective cohort study of children tested for SARS-CoV-2 infection, 0.67% and 0.16% of children with positive tests and negative tests met the WHO pediatric PCC definition at 12-month follow-up.^12^ In general, QoL as reported by participants at 6 and 12 months did not differ according to SARS-CoV-2 index visit test status. The most common symptoms reported by children with positive SARS-CoV-2 tests with PCC at 12 months were respiratory (eg, recurrent infections and congestion).

The absolute prevalence of PCC in children with positive SARS-CoV-2 tests in our study at 6 and 12 months were lower than earlier estimates.^4,9,10,26^ However, our findings align with studies that focus on the difference in the prevalence between children with positive tests and those with negative tests (ie, control group).^8,26,27^ Our methods, which included active approaches to minimize loss to follow-up, evaluation of PCC over a prolonged follow-up period, and the use of a PCC definition which incorporates QoL measures, represent advances that enhance the accuracy of our estimates. Prior studies with higher PCC estimates often only required the presence of ongoing symptoms,^10,26,28,29,30^ minimal changes in QoL,^9,26^ and did not require daily functioning to be inferior at follow-up to that preceding the acute infection.^9,10,26^ Our findings, however, may be less generalizable to older children, as over 80% of study participants were younger than 8 years and in general, younger children are at lower risk of reporting PCC.^31^ Interestingly, all PCC cases with SARS-CoV-2 positive tests were among children 8 years or younger. Additionally, although the PedsQL addresses issues of development indirectly, it lacks a dedicated developmental focus, which is an area targeted by the WHO PCC definition.^12^ As such, its use to define the presence or absence of a change in QoL may have led to an underestimation of PCC prevalence.

We found an increased prevalence of PCCs among children who had SARS-CoV-2 positive tests compared with the negative test control group. This finding is supported by the fact that a greater proportion of children with SARS-CoV-2 positive tests (17% and 24% at 6 and 12 months, respectively) compared with children with negative tests (15% at both time points) reported their child’s overall health was worse on a 0- to 100-point scale, compared with before the index ED visit illness. These results should be considered in the context of PedsQL findings, which did not differ between children with positive and negative tests at 6 and 12 months when analyzed as a continuous variable or when dichotomized using a reduced QoL cut point. These contradictory findings could reflect the subjective nature of reporting overall health on the 100-point scale as compared with completing individual QoL questions. This hypothesis is supported by evidence that prolonged symptoms appear to cluster within families^10,32,33^ and that the reporting of persistent physical symptoms is more strongly associated with the belief in having experienced COVID-19 than having laboratory-confirmed SARS-CoV-2 infection.^34^ These findings could reflect shared genetic vulnerability among family members leading to viral persistence or the possibility of an increased focus on ongoing symptoms in the presence of a symptomatic family member.^33^

Although this study is the first we know of to use the most recent WHO pediatric PCC definition,^12^ when long-term QoL has been evaluated in children with SARS-CoV-2 infection, similar conclusions have been reached. In a national, matched longitudinal cohort study of children conducted in England,^35^ several QoL and well-being measures revealed no differences between those with positive SARS-CoV-2 tests and those with negative tests at 6 and 12 months. This study unfortunately did not classify the presence or absence of PCC in study participants. In a Norwegian study that used the PedsQL to assess QoL in individuals aged 12 to 25 years tested for SARS-CoV-2 infection early in the pandemic; compared with our results, the authors reported much lower scores for both groups of participants (ie, those with positive and negative SARS-CoV-2 tests) at 6-month follow-up (medians of 78 and 76, respectively).^26^ As a less stringent version of the WHO PCC definition was used in that study,^26^ the authors reported a much higher PCC prevalence (49% in the cohort with positive tests and 47% in the cohort with negative tests). Our report builds on these studies by suggesting that overall QoL is not reduced by SARS-CoV-2 infection, and that PCC in children is uncommon.

### Limitations

This study has some limitations. Although 30% of consented eligible participants were lost to follow-up and those who completed follow-up were more likely to be infected by the Omicron variant, which is less strongly associated with the development of PCC,^36^ our data yield insights into the strain that is currently circulating. Our data collection relied on caregiver report, which has the potential to underreport or overreport symptoms, particularly for younger children and infants, and we relied on retrospective reporting of pre–SARS-CoV-2 testing health status, which is prone to recall bias. Although this may be important, as baseline overall health status was lower among those with negative tests relative to participants with positive tests, our sensitivity analysis findings revealed that this had a limited impact on our findings.

As we were unable to identify factors independently associated with risk of PCC due to the small number of events, we cannot conclude that SARS-CoV-2 test status is independently associated with PCC. We did not perform antibody testing to confirm the absence of SARS-CoV-2 infection during the study period in control participants, and thus control group contamination could have occurred, which would minimize our ability to detect between-group differences. This is an important consideration as just over one-half of unvaccinated children had SARS-CoV-2 spike antibodies indicative of infection and/or vaccination following the peak of the Omicron wave.^37^ Additionally, as illness severity may be different among children with SARS-CoV-2 who visit the ED and those who do not, generalizing our findings to the latter population should be performed with caution. However, as illness severity is associated with PCC,^8^ children not requiring ED evaluation likely have a lower PCC prevalence than we report.

## Conclusions

In this study, although few children had PCC at 12 months, the prevalence was greater among SARS-CoV-2 infected children compared with controls. The likelihood of having symptoms that reduce daily functioning was 0.5% greater among those who tested positive for SARS-CoV-2 infection compared with those who tested negative. QoL did not differ according to SARS-CoV-2 test status. The most common symptoms reported by children with positive SARS-CoV-2 tests with PCC at 12 months were respiratory.

## Notes

Supplement 1. **eTable 1. **Participating Study Sites and the Number of SARS-CoV-2 Positive and Negative Participants Included in the 12-Month Outcome Analysis **eTable 2. **Modified Version of the International Severe Acute Respiratory and Emerging Infection Consortium Questionnaire Administered at 6- and 12-Month Follow-Up **eTable 3. **Missing Data Among Study Participants Who Were Included in the Post–COVID-19 Condition Analysis at 12-Months **eTable 4. **Comparisons of Study Participants According to the Outcome of the Post-COVID Condition at 12 Months, Stratified by SARS-CoV-2 Acute Illness Status **eTable 5. **Comparison of Characteristics of Participants Who Reported Post-COVID Condition (PCC) Symptoms at 90-Day Follow-Up According to Our Ability to Classify the Presence/Absence of the PCC at 12-Month Follow-Up **eTable 6. **Comparison of Characteristics of Study Participants Who Completed 12-Month Follow-Up and Those Who Did Not **eTable 7. **Outcomes According to Index Emergency Department SARS-CoV-2 Test Result Status **eTable 8. **Summary of All Sensitivity Analyses Performed **eTable 9. **Reported History of Fever at Time of Follow-Up **eTable 10. **Symptoms at 6 and 12 Months According to Index Emergency Department Visit SARS-CoV-2 Status

Supplement 2.
Nonauthor Collaborators

Supplement 3.
Data Sharing Statement
