# Supplementary material for: Post–COVID-19 Condition in Children 6 and 12 Months After Infection
Source: JAMA Netw Open. 2023 Dec 28;6(12):e2349613. doi: 10.1001/jamanetworkopen.2023.49613 (PMC10755606; doi:10.1001/jamanetworkopen.2023.49613)
Supplement: Supplement 1. — eTable 1. Participating Study Sites and the Number of SARS-CoV-2 Positive and Negative Participants Included in the 12-Month Outcome Analysis eTable 2. Modified Version of the International Severe Acute Respiratory and Emerging Infection Consortium Questionnaire Administered at 6- and 12-Month Follow-Up eTable 3. Missing Data Among Study Participants Who Were Included in the Post–COVID-19 Condition Analysis at 12-Months eTable 4. Comparisons of Study Participants According to the Outcome of the Post-COVID Condition at 12 Months, Stratified by SARS-CoV-2 Acute Illness Status eTable 5. Comparison of Characteristics of Participants Who Reported Post-COVID Condition (PCC) Symptoms at 90-Day Follow-Up According to Our Ability to Classify the Presence/Absence of the PCC at 12-Month Follow-Up eTable 6. Comparison of Characteristics of Study Participants Who Completed 12-Month Follow-Up and Those Who Did Not eTable 7. Outcomes According to Index Emergency Department SARS-CoV-2 Test Result Status eTable 8. Summary of All Sensitivity Analyses Performed eTable 9. Reported History of Fever at Time of Follow-Up eTable 10. Symptoms at 6 and 12 Months According to Index Emergency Department Visit SARS-CoV-2 Status [file jamanetwopen-e2349613-s001.pdf]

Supplemental Online Content

Dun-Dery F, Xie J, Winston K, et al. Post–COVID-19 condition in children 6 and 12 months after infection. *JAMA Netw Open*. 2023;6(12):e2349613.
doi:10.1001/jamanetworkopen.2023.49613

eTable 1. Participating Study Sites and the Number of SARS-CoV- 2 Positive and Negative Participants Included in the 12-Month Outcome Analysis
eTable 2. Modified Version of the International Severe Acute Respiratory and Emerging Infection Consortium Questionnaire Administered at 6- and 12-Month Follow-Up
eTable 3. Missing Data Among Study Participants Who Were Included in the Post-COVID-19 Condition Analysis at 12-Months
eTable 4. Comparisons of Study Participants According to the Outcome of the Post-COVID-Condition§ at 12 Months, Stratified by SARS-CoV-2 Acute Illness Status
eTable 5. Comparison of Characteristics of Participants Who Reported Post-COVID Condition (PCC) Symptoms at 90-Day Follow Up According to Our Ability to Classify the presence/Absence of the PCC at 12-Month Follow-Up
eTable 6. Comparison of Characteristics of Study Participants Who Completed 12-Month Follow-Up and Those Who Did Not
eTable 7. Outcomes According to Index Emergency Department SARS-CoV-2 Test Result Status
eTable 8. Summary of All Sensitivity Analyses Performe**d**
eTable 9. Reported History of Fever at Time of Follow-Up
eTable 10. Symptoms at 6 and 12 Months According to Index Emergency Department Visit SARS-CoV-2 Status

This supplemental material has been provided by the authors to give readers additional information about their work.

© 2023 Dun-Dery F et al. *JAMA Network Open.*

eTable 1. Participating Study Sites and the Number of SARS-CoV- 2 Positive and Negative Participants Included in the 12-Month Outcome Analysis

	Total	SARS-CoV-2	
**Hospital Name (City)**		**Negative**	**Positive**
Montreal Children's Hospital (Montreal)	1289	1073	216
Alberta Children's Hospital (Calgary)	699	504	195
British Columbia Children's Hospital (Vancouver)	499	419	80
Centre Hospitalier Universitaire Sainte-Justine (Montreal)	467	261	206
Izaak Walton Killam Children's Health Centre (Halifax)	364	335	29
The Children's Hospital of Winnipeg (Winnipeg)	363	337	26
Children's Hospital of Eastern Ontario (Ottawa)	358	253	105
Centre Hospitalier de l'Université Laval (Quebec City)	355	272	83
McMaster Children's Hospital (Hamilton)	343	304	39
The Hospital for Sick Children (Toronto)	293	108	185
Jim Pattison Children's Hospital (Saskatoon)	237	215	22
Janeway Children's Health and Rehabilitation Centre (St. John's)	187	185	2
Children's Hospital London Health Sciences Centre (London)	95	91	4
Kingston Health Sciences Centre (Kingston)	14	14	0
**Total**	**5563**	**4371**	**1192**

© 2023 Dun-Dery F et al. *JAMA Network Open.*

eTable 2. Modified Version of the International Severe Acute Respiratory and Emerging Infection Consortium Questionnaire Administered at 6- and 12-Month Follow-Up

Long-COVID Questionnaire - ISARIC WHO COVID-19	
Has your child ever sought support from child /adolescent Mental Health services (psychologist, psychiatrist, social worker, counsellor) before the Covid-19 pandemic?<br><br>Votre enfant a-t-il déjà demandé un soutien auprès des services de santé mentale pour enfants/adolescents (psychologue, psychiatre, travailleur social, conseiller) avant la pandémie de Covid-19 ?	○ Yes // Oui<br>○ No // Non
Prior to your child coming to the emergency department on approximately [date_screen] how was your child's physical health in general?<br><br>Avant que votre enfant ne se présente aux urgences, comment était la santé physique de votre enfant en général ?	○ Very poor // Très mauvaise (noter la réponse dans la section commentaire)<br>○ Poor // Mauvaise (noter la réponse dans la section commentaire)<br>○ Ok // ok<br>○ Good // Bonne<br>○ Very good // Très bonne
If you chose poor or very poor, please explain:<br><br>(noter la réponse dans la section commentaire)	__________________________________
Prior to your child coming to the emergency department on approximately [date_screen], how would you describe your child's mental /psychological health in general<br><br>Avant que votre enfant ne se présente aux urgences, comment décririez-vous la santé mentale/psychologique de votre enfant en général	○ Very poor // Très mauvaise (noter la réponse dans la section commentaire)<br>○ Poor // Mauvaise (noter la réponse dans la section commentaire)<br>○ Ok // ok<br>○ Good // Bonne<br>○ Very good // Très bonne
If you chose poor or very poor, please explain:<br><br>(noter la réponse dans la section commentaire)	__________________________________

2023-06-22 17:02 projectredcap.org REDCap

© 2023 Dun-Dery F et al. *JAMA Network Open.*

Has your child visited a doctor/health center because of health consequences related to their emergency department visit on approximately [date_screen]?<br><br>Votre enfant a-t-il consulté un médecin/centre de santé en raison de conséquences sur sa santé liées à sa visite aux urgences?	◯ Yes // Oui<br>◯ No // Non
Has your child felt feverish recently?<br><br>Votre enfant s'est-il senti fiévreux récemment ?	◯ Yes // Oui<br>◯ No // Non
If yes indicate when you felt feverish<br><br>(tick all that apply)<br><br>Si oui, c'était quand ?	☐ Within the last 7 days // Au cours des 7 derniers jours<br>☐ 1-2 weeks // 1-2 semaines<br>☐ >2-4 weeks // 2-4 semaines<br>☐ >1-2 months // 1-2 mois<br>☐ >2-3 months // 2-3 mois<br>☐ >3-6 months // 3-6 mois<br>☐ Since illness onset bringing them to the emergency department // Depuis le début de la maladie les amenant aux urgences
If yes what was the most likely cause of your child's most recent feverish illness?<br><br>Si oui, quelle était la cause la plus probable de la dernière maladie fébrile de votre enfant ?	◯ COVID-19 // COVID-19<br>◯ Other respiratory infection (cough/cold/sore throat)<br>◯ TB // TB<br>◯ Stomach infection (diarrhea/vomiting) // Infection de l'estomac (diarrhée/vomissements)<br>◯ Urinary infection // Infection urinaire<br>◯ Other (Specify) // Autre (précisez)<br>◯ Unknown // Inconnu<br>◯ Prefer not to say // Préfère ne pas répondre
Please specify other<br><br>(précisez)	__________________________________
How much do you agree with the following statement?<br><br>"My child has fully recovered from the illness that brought them to the emergency department on approximately [date_screen]"<br><br>Please mark an X on the line below that match your opinion on the question as of TODAY:<br><br>ur une échelle de 0 (fortement en désaccord) à 10 (fortement d'accord) dans quelle mesure êtes-vous d'accord avec l'énoncé suivant ?<br><br>"Mon enfant s'est complètement rétablis de la maladie qui l'a amené aux urgences"	◯ 0 - Strongly Disagree<br>◯ 1<br>◯ 2<br>◯ 3<br>◯ 4<br>◯ 5 - Neutral<br>◯ 6<br>◯ 7<br>◯ 8<br>◯ 9<br>◯ 10 - Strongly Agree

© 2023 Dun-Dery F et al. *JAMA Network Open.*

How much difficulty is your child having breathing?

Please state the number under the figure that best describes it:

À quel point votre enfant a-t-il de la difficulté à respirer ?

- ◯ 1 - No trouble at all // Pas de problème du tout
- ◯ 2 - A tiny bit // Un tout petit peu
- ◯ 3 - A little // Un petit peu
- ◯ 4 - Some // Un peu
- ◯ 5 - Quite a bit // Plus qu'un peu
- ◯ 6 - A lot // Beaucoup
- ◯ 7 - Very much trouble // Énormément de problème

Within the last seven days, has your child had any of these symptoms, which were NOT present before the illness that brought your child coming to the emergency department on approximately [date_screen]? (Indicate if you have a symptom (tick Yes) and also if your child does not have a specific symptom (tick no))

Au cours des 7 derniers jours, votre enfant a-t-il présenté ... ? Si oui, ce symptôme était-il présent avant la visite à l'urgence de votre enfant ?

	Yes // Oui	No // Non	Unknown // Inconnu
Nasal congestion/rihnorrhea // Écoulement/ congestion nasale	◯	◯	◯
Difficulty breathing/chest tightness/breathlessness // Difficulté à respirer/ oppression thoracique/essoufflement	◯	◯	◯
Pain on breathing // Douleur à la respiration	◯	◯	◯
Chest pain // Douleur thoracique	◯	◯	◯
Persistent cough // Toux persistante	◯	◯	◯
Cannot fully move or control movement // Ne peut pas complètement bouger ou contrôler ses mouvements	◯	◯	◯
Problems with balance // Problèmes d'équilibre	◯	◯	◯
Persistent muscle pain // Douleur musculaire persistante	◯	◯	◯
Joint pain or joint swelling // Douleurs articulaires ou enflure	◯	◯	◯
Headache // Maux de tête	◯	◯	◯
Dizziness/light headedness // Étourdissements	◯	◯	◯
Fainting/blackouts // Évanouissements	◯	◯	◯

© 2023 Dun-Dery F et al. *JAMA Network Open.*

Problems seeing/blurred vision // Problèmes de vision/vision floue	○	○	○
Disturbed smell // Altération de l'odorat	○	○	○
Disturbed taste // Altération du goût	○	○	○
Tremor/shakiness // Tremblements	○	○	○
Tingling feeling/"pins and needles" // Sensation de picotement (aiguilles)	○	○	○
Seizures/fits // Convulsions	○	○	○
Confusion/lack of concentration // Confusion/manque de concentration	○	○	○
Problems speaking or communicating // Problèmes d'élocution ou de communication	○	○	○
Insomnia (hard to fall asleep, hard to stay asleep) // Insomnie (difficile de s'endormir, difficile de rester endormi)	○	○	○
Hypersomnia (excessive daytime sleepiness or prolonged nighttime sleep) // Hypersomnie (somnolence diurne excessive ou sommeil nocturne prolongé)	○	○	○
Fatigue // Fatigue	○	○	○
Weight loss // Perte de poids	○	○	○
Problems swallowing or chewing // Problèmes de déglutition ou de mastication	○	○	○
Poor appetite // Perte d'appétit	○	○	○
Diarrhea // Diarrhée	○	○	○
Stomach/abdominal pain // Douleur abdominale	○	○	○
Feeling nauseous // Nausées	○	○	○
Vomiting // Vomissements	○	○	○
Constipation // Constipation	○	○	○
Palpitations (heart racing) // Palpitations (accélération du rythme cardiaque)	○	○	○

© 2023 Dun-Dery F et al. *JAMA Network Open.*

Variations in heart rate (Tachycardia or bradycardia) // Variations de la fréquence cardiaque (Tachycardie ou bradycardie)	◯	◯	◯
Urination problems // Problèmes de miction (difficulté à uriner)	◯	◯	◯
Bilateral (both eyes) conjunctivitis (red or injected) // Conjonctivite bilatérale (les deux yeux) (rouge ou injectée)	◯	◯	◯
Skin rash // Éruption cutanée (plaques rouges sur le corps)	◯	◯	◯
Any other new symptoms, if yes, specify all: // Des nouveaux symptômes? Si oui, veuillez préciser:	◯	◯	◯

Changes in menstruation (if regular before illness that brought your child coming to the emergency department on approximately [date_screen])<br><br>Changements dans les menstruations (si régulières avant la maladie qui a amené votre enfant aux urgences) (poser la question seulement aux filles de plus de 8 ans)	◯ Yes // Oui<br>◯ No // Non<br>◯ Unknown // Inconnu<br>◯ Not Applicable
Persistent cough<br><br>Toux persistante	◯ dry cough // Sans sécrétions (toux sèche)<br>◯ with phlegm // Avec sécrétions (toux grasse)
Bilateral (both eyes) conjunctivitis (red or injected)	◯ Purulent<br>◯ Non-purulent
Skin Rash: If yes, tick all body areas affected	☐ face<br>☐ trunk (stomach or back)<br>☐ arms<br>☐ legs<br>☐ other
Skin Rash: If yes, tick all body areas affected<br><br>Specify other area	__________________________________
Any other new symptoms, if yes, specify all:	____

© 2023 Dun-Dery F et al. *JAMA Network Open.*

Since the illness that brought your child coming to the emergency department on approximately [date_screen], has your child been diagnosed with any of the following?

Depuis votre visite à l'urgence, votre enfant a-t-il reçu un nouveau diagnostique de ... ?

	Yes // Oui	No // Non	Unknown // Inconnu
Multisystem inflammatory syndrome of Children (MIS-C) // Syndrome inflammatoire multi systémique de l'enfant (MIS-C)	○	○	○
Pulmonary embolism/micro emboli (PE, Clot in lung) // Embolie pulmonaire/micro embolie (EP, caillot dans le poumon)	○	○	○
Kawasaki disease // Maladie de Kawasaki	○	○	○
Respiratory failure // Insuffisance respiratoire	○	○	○
Asthma // Asthme	○	○	○
Reduced lung function // Fonction pulmonaire réduite	○	○	○
Myocarditis (Inflammation of the heart muscle) // Myocardite (inflammation du muscle cardiaque)	○	○	○
Depression // Dépression	○	○	○
Anxiety // Anxiété	○	○	○
Diabetes // Diabète	○	○	○
Shock/toxic shock syndrome // Syndrome de choc/choc toxique	○	○	○
Coagulopathy (excessive bleeding or clotting) // Coagulopathie (saignement excessif ou coagulation)	○	○	○
Kidney problems // Problèmes rénaux	○	○	○
Intussusception // Intussusception (invagination intestinale)	○	○	○
Other (please indicate) // Autre (veuillez préciser)	○	○	○

Diabetes (if yes indicate type):
○ Type 1
○ Type 2

Other (please indicate)

____

© 2023 Dun-Dery F et al. *JAMA Network Open.*

Your child's overall health status
We would like to know how good or bad your child's health is
- Line is numbered 0 to 100
- 100% means the best health you can imagine
- 0% means the worst health you can imagine
- Please mark an X on the line that shows how good or bad your child's health is TODAY and how it was BEFORE their COVID-19 illness

Quel est l'état de santé général de votre enfant sur une échelle de 0 (la pire santé) à 100 (la meilleure santé) ?

Today<br><br>Aujourd'hui	The worst health you can imagine // Le pire état de santé possible — The best health you can imagine // = le meilleur état de santé possible<br>(Place a mark on the scale above)
Before your child's illness that brought them to the emergency department<br><br>Avant sa visite à l'urgence	The worst health you can imagine // Le pire état de santé possible — The best health you can imagine // le meilleur état de santé possible<br>(Place a mark on the scale above)

Health and wellbeing For Children and Teenagers from 8 to 17 years old
If you/your child is under 8 years old skip to question 10.

Child less than 8 years old	○ Less than 8 years old
Describe your child's health TODAY	
Mobility (walking about)	○ I have no problems walking about<br>○ I have some problems walking about<br>○ I have a lot of problems walking about
Looking after myself	○ I have no problems washing or dressing myself<br>○ I have some washing or dressing myself<br>○ I have a lot of problems washing or dressing myself
Doing usual activities (for example going to school, hobbies, sports, playing, doing things with family or friends)	○ I have no problems doing my usual activities<br>○ I have some problems doing my usual activities<br>○ I have a lot of problems doing my usual activities
Having pain or discomfort	○ I have no pain or discomfort<br>○ I have some pain or discomfort<br>○ I have a lot of pain or discomfort
Feeling worried, sad or unhappy	○ I am not worried, sad or unhappy<br>○ I am a bit worried, sad or unhappy<br>○ I am very worried sad or unhappy

© 2023 Dun-Dery F et al. *JAMA Network Open.*

Describe your child's health BEFORE onset of the illness that brought your child the emergency department on approximately [date_screen].	
Mobility (walking about)	◯ I had no problems walking about<br>◯ I had some problems walking about<br>◯ I had a lot of problems walking about
Looking after myself	◯ I had no problems washing or dressing myself<br>◯ I had some washing or dressing myself<br>◯ I had a lot of problems washing or dressing myself
Doing usual activities (for example going to school, hobbies, sports, playing, doing things with family or friends)	◯ I had no problems doing my usual activities<br>◯ I had some problems doing my usual activities<br>◯ I had a lot of problems doing my usual activities
Having pain or discomfort	◯ I had no pain or discomfort<br>◯ I had some pain or discomfort<br>◯ I had a lot of pain or discomfort
Feeling worried, sad or unhappy	◯ I was not worried, sad or unhappy<br>◯ I was a bit worried, sad or unhappy<br>◯ I was very worried sad or unhappy
Please let us know of any additional comments about your child's illness:	______________________________

© 2023 Dun-Dery F et al. *JAMA Network Open.*

eTable 3. Missing Data Among Study Participants Who Were Included in the Post-COVID-19 Condition* Analysis at 12-Months

Characteristics	SARS-CoV-2 Test Result		
	All (5563)	SARS-CoV-2 Positive (n=1192)	SARS-CoV-2 Negative (n=4371)
Age	0 (0)	0 (0)	0 (0)
Sex, male, n (%)	0 (0)	0 (0)	0 (0)
Chronic underlying condition, n (%)	5 (0.89)	3 (0.25)	2 (0.05)
History of asthma, n (%)	4 (0.07)	3 (0.25)	1 (0.02)
Variant of concern, n (%)	0 (0)	0 (0)	N/A
Hospitalization, n (%)†	0 (0)	0 (0)	0 (0)
ICU admission, n (%)†	0 (0)	0 (0)	0 (0)
Antibiotics, n (%)†	21 (0.38)	5 (0.42)	16 (0.37)
Corticosteroids, n (%)†	24 (0.43)	10 (0.84)	14 (0.32)
Number of symptoms at the index ED visit	3 (0.05)	2 (0.17)	1 (0.02)

ED: Emergency Department; ICU: Intensive Care Unit

†: At or within 14 days of the index ED visit.

§ For SARS-CoV-2 negative participants, use of the PCC term refers to meeting the symptom and quality of life aspects of the definition but excludes the requirement to test positive for SARS-CoV-2 nucleic acid.

© 2023 Dun-Dery F et al. *JAMA Network Open.*

eTable 4. Comparisons of Study Participants According to the Outcome of the Post-COVID-Condition§ at 12 Months, Stratified by SARS-CoV-2 Acute Illness Status

Characteristics	All (5563)	SARS-CoV-2 Test Result					
		SARS-CoV-2 Positive (n=1192)			SARS-CoV-2 Negative (n=4371)		
		Did Not Develop PCC n=1184	Developed PCC n=8	Adjusted P Value¶	Did Not Develop PCC n=4364	Developed PCC n=7	Adjusted P Value¶
Age (year), median (IQR)	2.0 (0.9, 5.0)	2.0 (0.5, 7.0)	2.5 (0.4, 7.0)	0.99	2.0 (1.0, 5.0)	2.0 (0.7, 4.0)	0.99
Sex, male, n (%)	2956/5563 (53.1)	658/1184 (55.6)	3/8 (37.5)	0.99	2292/4364 (52.5)	3/7 (42.9)	0.99
Chronic underlying condition (excluding asthma), n (%)	722/5558 (13.0)	180/1181 (15.2)	1/8 (12.5)	>0.99	539/4362 (12.4)	2/7 (28.6)	0.80
History of asthma, n (%)	522/5559 (9.4)	82/1181 (6.9)	1/8 (12.5)	>0.99	438/4363 (10.0)	1/7 (14.3)	0.99
Received COVID Vaccination prior to enrollment, n (%)⁑				>0.99			>0.99
No	2570/2917 (88.1)	684/832 (82.2)	7/7 (100)		1875/2074 (90.4)	4/4 (100)	
Yes*	170/2917 (5.8)	69/832 (8.3)	0/7 (0)		101/2074 (4.9)	0/4 (0)	
Unknown	177/2917 (6.1)	79/832 (9.5)	0/7 (0)		98/2074 (4.7)	0/4 (0)	
Number of symptoms at the index ED visit, median (IQR)	6.0 (4.0, 8.0	6.0 (3.0, 8.3) n=1182	8.0 (7.0, 9.0) n=8	0.66	6.0 (4.0, 8.0) n=4363	9.0 (6.0, 12.0) n=7	0.66
Variant of concern, n (%)				0.82			
Wild type	322/1192 (27.0)	321/1184 (27.1)	1/8 (12.5)		n/a	n/a	
Alpha	189/1192 (15.9)	189/1184 (16)	0/8 (0)		n/a	n/a	
Gamma	4/1192 (0.3)	4/1184 (0.3)	0/8 (0)		n/a	n/a	
Delta	259/1192 (21.7)	255/1184 (21.5)	4/8 (50)		n/a	n/a	
Omicron	418/1192 (35.1)	415/1184 (35.1)	3/8 (37.5)		n/a	n/a	

© 2023 Dun-Dery F et al. *JAMA Network Open.*

Characteristics (continued)	All (5563) (continued)	SARS-CoV-2 test Results (continued)					
		SARS-CoV-2 Positive (n=1192)			SARS-CoV-2 Negative (n=4371)		
		Did Not Develop PCC n=1184	Developed PCC n=8	Adjusted P Value¶	Did Not Develop PCC n=4364	Developed PCC n=7	Adjusted P Value¶
Hospitalization, n (%)†	543/5563 (9.8)	134/1184 (11.3)	2/8 (25.0)	0.80	406/4364 (9.3)	1/7 (14.3)	0.99
ICU admission, n (%)†	36/5563 (0.6)	8/1184 (0.7)	0/8 (0)	>0.99	28/4364 (0.6)	0/7 (0)	>0.99
Antibiotics, n (%)†	1008/5542 (18.2)	171/1179 (14.5)	1/8 (12.5)	>0.99	833/4348 (19.2)	3/7 (42.9)	0.71
Corticosteroids, n (%)†	853/5539 (15.4)	147/1174 (12.5)	3/8 (37.5)	0.66	702/4350 (16.1)	1/7 (14.3)	>0.99

ED: Emergency Department; ICU: Intensive Care Unit; N/A: Not Applicable.

*Yes = had received at least 1 dose of any approved COVID vaccine.

†: At or within 14 days of the index ED visit.

*: some participants did not have vaccination data because they were enrolled before the vaccination question was added to the study questionnaire (June 11, 2021).

¶: P values were adjusted for multiple comparisons via Benjamini-Hochberg method.

§ For SARS-CoV-2 negative participants, use of the PCC term refers to meeting the symptom and quality of life aspects of the definition but excludes the requirement to test positive for SARS-CoV- 2 nucleic acid.

© 2023 Dun-Dery F et al. *JAMA Network Open.*

eTable 5. Comparison of Characteristics of Participants Who Reported Post-COVID Condition (PCC) Symptoms at 90-Day Follow Up According to Our Ability to Classify the presence/Absence of the PCC at 12-Month Follow-Up

Characteristics	PCC Classified at 12-Months (n=285)	PCC Not Classified at 12-Months (n=298)
Age, years, median (IQR)	2.0 (1.0, 4.0)	1.3 (0.9, 6.0)
Sex, male, n (%)	150/285 (52.6)	164/298 (55.0)
Chronic underlying condition, n (%)	42/284 (14.8)	38/298 (12.8)
History of asthma, n (%)	38/284 (13.4)	36/298 (12.1)
SARS-CoV-2 positive	81/285 (28.4)	74/298 (24.8)
Variant of concern, n (%)		
Wild type	8/81 (9.9)	34/74 (45.9)
Alpha	19/81 (23.5)	13/74 (17.6)
Beta	0/81 (0)	0/74 (0)
Gamma	0/81 (0)	1/74 (1.4)
Delta	26/81 (32.1)	18/74 (24.3)
Omicron	28/81 (34.6)	8/74 (10.8)
Hospitalization, n (%)†	39/285 (13.7)	48/298 (16.1)
ICU admission, n (%)†	4/285 (1.4)	3/298 (1.0)
Antibiotics, n (%)†	57/284 (20.1)	74/298 (24.8)
Corticosteroids, n (%)†	77/284 (27.1)	73/298 (24.5)
Number of symptoms at the index ED visit	7.0 (4.0, 9.0)	8.0 (5.0, 10.0)

ED: Emergency Department; ICU: Intensive Care Unit; PCC: Post-COVID Condition.
†: At or within 14 days of the index ED visit.

© 2023 Dun-Dery F et al. *JAMA Network Open.*

eTable 6. Comparison of Characteristics of Study Participants Who Completed 12-Month Follow-Up and Those Who Did Not

Characteristics	Did Not Complete 12-Month Follow-Up (n=1364)	Completed 12-Month Follow-Up (n=3256)
Age, years, median (IQR)	2.0 (0.9, 5.0)	2.0 (0.9, 5.0)
Sex, male, n (%)	748/1364 (54.8)	1702/3256 (52.3)
Chronic underlying condition, n (%)	187/1362 (13.7)	413/3251 (12.7)
History of asthma, n (%)	134/1362 (9.8)	292/3252 (9.0)
Had PCC at 90-day follow-up	94/943 (10.0)	286/2686 (10.6)
SARS-CoV-2 positive	402/1364 (29.5)	731/3256 (22.5)
Variant of concern, n (%)		
Wild type	147/402 (36.6)	106/731 (14.5)
Alpha	96/402 (23.9)	98/731 (13.4)
Beta	1/402 (0.2)	0/731 (0)
Gamma	6/402 (1.5)	0/731 (0)
Delta	78/402 (19.4)	179/731 (24.5)
Omicron	74/402 (18.4)	348/731 (47.6)
Hospitalization, n (%)†	163/1364 (12.0)	330/3256 (10.1)
ICU admission, n (%)†	4/1364 (0.3)	23/3256 (0.7)
Antibiotics, n (%)†	243/1358 (17.9)	603/3245 (18.6)
Corticosteroids, n (%)†	203/1356 (15.0)	518/3243 (16.0)
Number of symptoms at the index ED visit	6.0 (4.0, 9.0)	6.0 (4.0, 8.0)

ED: Emergency Department; ICU: Intensive Care Unit; PCC: Post-COVID Condition.

†: At or within 14 days of the index ED visit.

© 2023 Dun-Dery F et al. *JAMA Network Open.*

eTable 7. Outcomes According to Index Emergency Department SARS-CoV-2 Test Result Status†

A. Male participants

Elements Defining the Composite Post-COVID Condition Outcome Measure	SARS-CoV-2 Positive (N=661)	SARS-CoV-2 Negative (N=2295)	Absolute Difference (95%CI of Difference)
**12-Month Follow-Up Survey Outcome Data**			
Met PCC definition§	3/661 (0.45)	3/2295 (0.13)	0.32 (-0.18, 1.0)
Any chronic signs/symptoms or diagnoses at 30 – 90 days following index ED visit, n (%)	46/598 (7.7)	104/2065 (5.0)	2.7 (0.3, 5.0)
Any chronic signs/symptoms or diagnoses at 9 – 13 months following index ED visit, n (%)	41/379 (10.8)	143/1316 (10.9)	-0.05 (-3.6, 3.5)
Overall health status at the time of the index ED visit as rated by caregivers on a 0-to-100-point scale at the time of 12-month follow-up data collection, median (IQR)¶	94.0 (75.0, 100) n=375	89.0 (60.0, 100) n=1306	5.0 (1.9, 8.1)
Overall health status at the time of 12-month follow-up as rated by caregivers on a 0-to-100-point scale at the time of 12-month follow-up data collection, median (IQR)¶	95.0 (85.0, 100) n=376	95.0 (85.0, 100) n=1310	0 (-2.4, 2.4)
Overall health status at 12-month was rated as lower than prior to the index illness, n (%)	97/375 (25.9)	195/1306 (14.9)	10.9 (6.1, 15.8)
PedsQL™ score (age > 2 years), median (IQR)*	97.9 (88.4, 100) n=243	97.9 (91.25, 100) n=1028	0 (-1.7, 1.7)
PedsQL™ score (age > 2 years) <78.6, n (%)*	26/243 (10.7)	82/1028 (8.0)	2.7 (-1.5, 6.9)
**6-Month Follow-Up Survey Outcome Da6ta**			
	**SARS-CoV-2 Positive (n=640)**	**SARS-CoV-2 Negative (n=2107)**	**Absolute Difference (95%CI of Difference)**

© 2023 Dun-Dery F et al. *JAMA Network Open.*

Met PCC definition§	4/640 (0.63)	1/2107 (0.047)	0.58 (0.13, 1.5)
Any chronic signs/symptoms or diagnoses at 30 – 90 days following index ED visit, n (%)	37/589 (6.3)	53/2014 (2.6)	3.7 (1.6, 5.7)
Any chronic signs/symptoms or diagnoses at 3 – 6 months following index ED visit, n (%)	24/282 (8.5)	64/578 (11.1)	-2.6 (-6.7, 1.6)
Overall health status at the time of the index ED visit as rated by caregivers on a 0-to-100-point scale at the time of 6-month follow-up data collection, median (IQR)	90.0 (60.0, 100) n=279	88.5 (60.0, 99.3) n=574	1.5 (-1.5, 5.5)
Overall health status at the time of 6-month follow-up as rated by caregivers on a 0-to-100-point scale at the time of 6-month follow-up data collection, median (IQR)	98.0 (90.0, 100) n=279	95.0 (86.0, 100) n=575	3.0 (0.3, 5.7)
Overall health status at 6-month was rated as lower than prior to the index illness, n (%)	43/279 (15.4)	82/574 (14.3)	1.1 (-4.0, 6.2)
PedsQL™ score (age > 2 years), median (IQR)	100 (91.1, 100) n=164	100 (92.5, 100) n=370	0 (-1.4, 1.4)
PedsQL™ score (age > 2 years) <78.6, n (%)	17/164 (10.4)	24/370 (6.5)	3.9 (-1.4, 9.2)

CI: Confidence Interval; ED: Emergency Department; IQR: interquartile range; PedsQL™: Pediatric Quality of Life Inventory™, Generic Core Scale; PCC: Post-COVID-19 Condition.

† Although we were able to classify the presence/absence of the Post-COVID-19 Condition (PCC) for 1192 SARS-CoV-2 positive children and 4371 SARS-CoV-2 negative children at 12-months (and 1152 and 3995, respectively, at 6-months) as described in the Methods section, not all sub-elements of the PCC diagnostic criteria are required to permit classification. Thus, although 1192 SARS-CoV-2 positive and 4371 SARS-CoV-2 negative children had the presence of PCC classified at 12-months, individual element results are reported for varying numbers of participants.

* n=456 of the SARS-CoV-2 positive study participants and n=1951 of the SARS-CoV-2 negative study participants > 2 years for the PedsQL™ score. The reported score was the mean of the total sores of the 5 domains.

P values were adjusted for multiple comparisons via Benjamini-Hochberg method.

§ For SARS-CoV-2 negative participants, use of the PCC term refers to meeting the symptom and quality of life aspects of the definition, but excludes the requirement to test positive for SARS-CoV- 2 nucleic acid.

© 2023 Dun-Dery F et al. *JAMA Network Open.*

7B. Female participants

Elements Defining the Composite Post-COVID Condition Outcome Measure	SARS-CoV-2 Positive (N=531)	SARS-CoV-2 Negative (N=2076)	Absolute Difference (95%CI of Difference)
**12-Month Follow-Up Survey Outcome Data**			
Met PCC definition§	5/531 (0.94)	4/2076 (0.19)	0.75 (0.10, 1.9)
Any chronic signs/symptoms or diagnoses at 30 – 90 days following index ED visit, n (%)	35/491 (7.1)	100/1850 (5.4)	1.7 (1.3, 4.2)
Any chronic signs/symptoms or diagnoses at 9 – 13 months following index ED visit, n (%)	30/348 (8.6)	124/1201 (10.3)	-1.7 (-5.1, 1.7)
Overall health status at the time of the index ED visit as rated by caregivers on a 0-to-100-point scale at the time of 12-month follow-up data collection, median (IQR)¶	95.0 (80.0, 100) n=345	89.0 (60.0, 100) n=1189	6.0 (2.4, 9.6)
Overall health status at the time of 12-month follow-up as rated by caregivers on a 0-to-100-point scale at the time of 12-month follow-up data collection, median (IQR)¶	97.0 (88.0, 100) n=347	95.0 (90.0, 100) n=1194	2.0 (-0.6, 4.6)
Overall health status at 12-month was rated as lower than prior to the index illness, n (%)	73/345 (21.2)	179/1189 (15.1)	6.1 (1.3, 10.9)
PedsQL™ score (age > 2 years), median (IQR)*	98.8 (91.5, 100) n=213	100 (91.7, 100) n=923	-1.3 (-2.3, -0.3)
PedsQL™ score (age > 2 years) <78.6, n (%)*	25/213 (11.7)	77/923 (8.3)	3.4 (-1.3, 8.1)
**6-Month Follow-Up Survey Outcome Da6ta**			
	**SARS-CoV-2 Positive (n=512)**	**SARS-CoV-2 Negative (n=1888)**	**Absolute Difference (95%CI of Difference)**

© 2023 Dun-Dery F et al. *JAMA Network Open.*

Met PCC definition§	2/512 (0.39)	3/1888 (0.23)	0.23 (-0.32, 1.1)
Any chronic signs/symptoms or diagnoses at 30 – 90 days following index ED visit, n (%)	30/486 (6.2)	44/1794 (2.5)	3.7 (1.5, 6.0)
Any chronic signs/symptoms or diagnoses at 3 – 6 months following index ED visit, n (%)	11/224 (4.9)	45/526 (8.6)	-3.6 (-7.2, 0.4)
Overall health status at the time of the index ED visit as rated by caregivers on a 0-to-100-point scale at the time of 6-month follow-up data collection, median (IQR)	94.0 (54.0, 100) n=222	90.0 (52.8, 100) n=518	3.0 (-3.0, 9.0)
Overall health status at the time of 6-month follow-up as rated by caregivers on a 0-to-100-point scale at the time of 6-month follow-up data collection, median (IQR)	100 (90.0, 100) n=223	95.0 (87.0, 100) n=523	5.0 (2.4, 7.6)
Overall health status at 6-month was rated as lower than prior to the index illness, n (%)	41/222 (18.5)	77/518 (14.9)	3.6 (-2.3, 9.6)
PedsQL™ score (age > 2 years), median (IQR)	100 (92.5, 100) n=118	100 (95.0, 100) n=336	0 (-1.0, 1.0)
PedsQL™ score (age > 2 years) <78.6, n (%)	8/118 (6.8)	24/336 (7.1)	-0.4 (-5.4, 5.6)

CI: Confidence Interval; ED: Emergency Department; IQR: interquartile range; PedsQL™: Pediatric Quality of Life Inventory™, Generic Core Scale; PCC: Post-COVID-19 Condition.

† Although we were able to classify the presence/absence of the Post-COVID-19 Condition (PCC) for 1192 SARS-CoV-2 positive children and 4371 SARS-CoV-2 negative children at 12-months (and 1152 and 3995, respectively, at 6-months) as described in the Methods section, not all sub-elements of the PCC diagnostic criteria are required to permit classification. Thus, although 1192 SARS-CoV-2 positive and 4371 SARS-CoV-2 negative children had the presence of PCC classified at 12-months, individual element results are reported for varying numbers of participants.

* n=456 of the SARS-CoV-2 positive study participants and n=1951 of the SARS-CoV-2 negative study participants > 2 years for the PedsQL™ score. The reported score was the mean of the total sores of the 5 domains.

P values were adjusted for multiple comparisons via Benjamini-Hochberg method.

§ For SARS-CoV-2 negative participants, use of the PCC term refers to meeting the symptom and quality of life aspects of the definition, but excludes the requirement to test positive for SARS-CoV- 2 nucleic acid.

© 2023 Dun-Dery F et al. *JAMA Network Open.*

eTable 8. Summary of All Sensitivity Analyses Performed

The following table summarizes the sensitivity analyses performed regarding evaluation of modifications to the definition of the Post-COVID Condition (PCC) employed as the primary outcome as well as to the population of participants included in the analyses. All analyses refer to the presence of the PCC based on data collected at the 12-month follow-up survey.

Supplementary Analysis	SARS-CoV-2 Positive	SARS-CoV-2 Negative	Difference	95%CI of the Difference
Timing of symptom onset removed from PCC definition	1.67%<br><br>20/1194	0.64%<br><br>28/4377	1.0%	0.3%, 1.8%
Children who were asymptomatic at the 90-day survey but failed to complete the 12-month survey were excluded from the study cohort	0.81%<br><br>8/984	0.23%<br><br>7/3109	0.6%	0.04%, 1.3%
Overall health status – reported as lower at 12-month than before at index illness removed from PCC definition	1.51%<br><br>18/1189	0.51%<br><br>22/4355	1.0%	0.3%, 1.7%

© 2023 Dun-Dery F et al. *JAMA Network Open.*

eTable 9. Reported History of Fever at Time of Follow-Up

	Total	SARS-CoV-2 Negative	SARS-CoV-2 Positive	P-Value
**At 6-Month Follow-Up**				
Any reported fever	493/1502 (32.8)	349/1039 (33.6)	144/463 (31.1)	0.34
Within the last 7 days	102/1502 (6.8)	74/1039 (7.1)	28/463 (6.0)	0.44
1-2 weeks	101/1502 (6.7)	75/1039 (7.2)	26/463 (5.6)	0.25
>2-4 weeks	138/1502 (9.2)	96/1039 (9.2)	42/463 (9.1)	0.92
>1-2 months	118/1502 (7.9)	92/1039 (8.9)	26/463 (5.6)	0.03
>2-3 months	81/1502 (5.4)	59/1039 (5.7)	22/463 (4.8)	0.46
>3-6 months	55/1502 (3.7)	47/1039 (4.5)	8/463 (1.7)	0.008
>6 months	2/1502 (0.1)	1/1039 (0.1)	1/463 (0.2)	0.52
**At 12-Month Follow-Up**				
Any reported fever	1406/3241 (43.4)	1068/2514 (42.5)	338/727 (46.5)	0.06
Within the last 7 days	301/3241 (9.3)	243/2514 (9.7)	58/727 (8.0)	0.17
1-2 weeks	286/3241 (8.8)	219/2514 (8.7)	67/727 (9.2)	0.67
>2-4 weeks	346/3241 (10.7)	272/2514 (10.8)	74/727 (10.2)	0.62
>1-2 months	370/3241 (11.4)	267/2514 (10.6)	103/727 (14.2)	0.008
>2-3 months	254/3241 (7.8)	187/2514 (7.4)	67/727 (9.2)	0.12
>3-6 months	228/3241 (7)	179/2514 (7.1)	49/727 (6.7)	0.72
>6 months	15/3241 (0.5)	8/2514 (0.3)	7/727 (1.0)	0.06

© 2023 Dun-Dery F et al. *JAMA Network Open.*

eTable 10. Symptoms at 6 and 12 Months According to Index Emergency Department Visit SARS-CoV-2 Status

A. Children Aged 0 to <8 Years

	Age 0 to < 8 Years			
	SARS-CoV-2 Positive		SARS-CoV-2 Negative	
	6 Month Follow-Up	12 Month Follow-Up	6 Month Follow-Up	12 Month Follow-Up
General/Systemic				
Fatigue	15/404 (3.7)	41/580 (7.1)	37/981 (3.8)	115/2154 (5.3)
Weight loss	8/404 (2.0)	19/580 (3.3)	14/981 (1.4)	38/2154 (1.8)
Poor appetite	17/404 (4.2)	48/580 (8.3)	55/981 (5.6)	136/2154 (6.3)
Hypersomnia	3/404 (0.7)	10/580 (1.7)	10/981 (1.0)	20/2153 (0.9)
Insomnia	10/404 (2.5)	38/580 (6.6)	34/981 (3.5)	96/2153 (4.5)
Disturbed smell	0/404 (0)	2/579 (0.3)	0/981 (0)	2/2153 (0.1)
Disturbed taste	0/404 (0)	2/580 (0.3)	3/981 (0.3)	1/2153 (0.05)
Skin rash	25/403 (6.2)	49/580 (8.4)	54/981 (5.5)	98/2155 (4.5)
Respiratory				
Nasal Congestion/Rhinorrhea	78/404 (19.3)	157/580 (27.1)	232/981 (23.6)	592/2155 (27.5)
Difficulty breathing/chest tightness/breathlessness	18/404 (4.5)	32/580 (5.5)	43/981(4.4)	128/2155 (5.9)
Pain on breathing	4/404 (1.0)	1/580 (0.2)	5/981 (0.5)	13/2154 (0.6)

© 2023 Dun-Dery F et al. *JAMA Network Open.*

**Persistent cough**	43/404 (10.6)	89/580 (15.3)	129/981 (13.1)	392/2154 (18.2)
**Problems swallowing or chewing**	3/404 (0.7)	5/580 (0.9)	8/981 (0.8)	19/2154 (0.9)
**Cardiac**				
**Chest pain**	3/404 (0.7)	3/580 (0.5)	4/981 (0.4)	15/2152 (0.7)
**Palpitations**	3/404 (0.7)	3/580 (0.5)	3/981 (0.3)	10/2153 (0.5)
**Variations in heart rate**	2/404 (0.5)	5/580 (0.9)	1/981 (0.1)	6/2153 (0.3)
**Musculoskeletal**				
**Persistent muscle pain**	6/404 (1.5)	7/580 (1.2)	7/981 (0.7)	26/2154 (1.2)
**Joint pain or joint swelling**	2/404 (0.5)	4/580 (0.7)	10/981 (1.0)	18/2154 (0.8)
**Gastrointestinal**				
**Diarrhea**	21/404 (5.2)	29/579 (5.0)	48/981 (4.9)	88/2154 (4.1)
**Stomach/abdominal pain**	13/404 (3.2)	25/580 (4.3)	33/981 (3.4)	69/2154 (3.2)
**Feeling nauseous**	6/404 (1.5)	13/579 (2.2)	16/981 (1.6)	47/2154 (2.2)
**Vomiting**	17/405 (4.2)	25/579 (4.3)	29/981 (3.0)	78/2154 (3.6)
**Constipation**	17/404 (4.2)	22/580 (3.8)	28/981 (2.9)	75/2154 (3.5)
**Neurologic**				
**Seizures/fits**	1/404 (0.2)	3/580 (0.5)	2/981 (0.2)	10/2154 (0.5)
**Fainting/blackouts**	0/404 (0)	1/580 (0.2)	0/981 (0)	4/2154 (0.2)
**Dizziness/light headedness**	4/404 (1.0)	4/580 (0.7)	0/981 (0)	8/2154 (0.4)

© 2023 Dun-Dery F et al. *JAMA Network Open.*

**Confusion/lack of concentration**	3/404 (0.7)	3/580 (0.5)	5/981 (0.5)	8/2153 (0.4)
**Problems speaking or communicating**	7/404 (1.7)	7/580 (1.2)	9/981 (0.9)	22/2153 (1.0)
**Headache**	10/404 (2.5)	14/580 (2.4)	24/981 (2.4)	58/2154 (2.7)
**Problems seeing/blurred vision**	0/404 (0)	0/580 (0)	2/981 (0.2)	3/2154 (0.1)
**Problems with balance**	1/404 (0.2)	3/580 (0.5)	2/981 (0.2)	20/2153 (0.9)
**Tremor/shakiness**	2/404 (0.5)	3/580 (0.5)	5/981 (0.5)	9/2153 (0.4)
**Cannot fully move or control movement**	1/405 (0.2)	1/580 (0.2)	0/981 (0)	7/2153 (0.3)
**Tingling feeling/"pins and needles"**	0/404 (0)	2/580 (0.3)	0/981 (0)	4/2153 (0.2)
**Miscellaneous**				
**Urination problems**	3/404 (0.7)	2/580 (0.3)	2/981 (0.2)	15/2153 (0.7)
**Bilateral (both eyes) conjunctivitis**	1/404 (0.2)	5/580 (0.9)	10/981 (1.0)	20/2153 (0.9)
**Changes in menstruation**	0/18 (0)	0/51	0/50 (0)	1/137 (0.7)

© 2023 Dun-Dery F et al. *JAMA Network Open.*

B. Children Aged 8 to <18 years

	Age 8 – < 18 Years			
	SARS-CoV-2 Positive		SARS-CoV-2 Negative	
	6 Month Follow-Up	12 Month Follow-Up	6 Month Follow-Up	12 Month Follow-Up
**General/Systemic**				
**Fatigue**	12/100 (12.0)	12/147 (8.2)	7/125 (5.6)	21/361 (5.8)
**Weight loss**	3/100 (3.0)	4/147 (2.7)	3/125 (2.4)	8/361 (2.2)
**Poor appetite**	8/100 (2.4)	8/147 (5.4)	8/125 (6.4)	17/361 (4.7)
**Hypersomnia**	2/100 (2.0)	4/147 (2.7)	4/125 (3.2)	11/361 (3.0)
**Insomnia**	5/100 (5.0)	12/147 (8.2)	4/125 (3.2)	17/361 (4.7)
**Disturbed smell**	2/100 (2.0)	1/147 (0.7)	0/125 (0)	4/361 (1.1)
**Disturbed taste**	2/100 (2.0)	2/147 (1.4)	0/125 (0)	2/361 (0.6)
**Skin rash**	5/100 (5.0)	4/147 (2.7)	2/125 (1.6)	9/361 (2.5)
**Respiratory**				
**Nasal Congestion/Rhinorrhea**	13/100 (13.0)	21/147 (14.3)	10/125 (8.0)	38/361 (10.5)
**Difficulty breathing/chest tightness/breathlessness**	7/100 (7.0)	6/147 (1.8)	6/125 (4.8)	14/361 (3.9)
**Pain on breathing**	1/100 (1.0)	1/147 (0.7)	1/125 (0.8)	6/361 (1.7)
**Persistent cough**	9/100 (9.0)	14/147 (9.5)	6/125 (4.8)	25/361 (6.9)
**Problems swallowing or chewing**	1/100 (1.0)	2/147 (1.4)	0/125 (0)	2/361 (0.6)
**Cardiac**				

© 2023 Dun-Dery F et al. *JAMA Network Open.*

**Chest pain**	2/100 (2.0)	5/147 (3.4)	1/125 (0.8)	6/361 (1.7)
**Palpitations**	1/100 (1.0)	1/147 (0.7)	2/125 (1.6)	3/361 (0.8)
**Variations in heart rate**	3/100 (3.0)	0/147 (0)	3/125 (2.4)	3/361 (0.8)
**Musculoskeletal**				
**Persistent muscle pain**	2/100 (2.0)	7/147 (4.8)	3/125 (2.4)	18/361 (5.0)
**Joint pain or joint swelling**	4/100 (4.0)	4/147 (2.7)	2/125 (1.6)	19/361 (5.3)
**Gastrointestinal**				
**Diarrhea**	3/100 (3.0)	6/146 (4.1)	3/125 (2.4)	8/361 (2.2)
**Stomach/abdominal pain**	6/100 (6.0)	12/147 (8.2)	8/125 (6.4)	17/361 (4.7)
**Feeling nauseous**	8/100 (8.0)	7/147 (4.8)	4/125 (3.2)	10/361 (2.8)
**Vomiting**	4/100 (4.0)	3/147 (2.0)	2/125 (1.6)	4/361 (1.1)
**Constipation**	1/100 (1.0)	6/147 (4.1)	6/125 (4.8)	8/361 (2.2)
**Neurologic**				
**Seizures/fits**	0/100 (0)	1/147 (0.7)	2/125 (1.6)	3/361 (0.8)
**Fainting/blackouts**	1/100 (1.0)	2/147 (1.4)	1/125 (0.8)	2/361 (0.6)
**Dizziness/light headedness**	4/100 (4.0)	8/147 (5.4)	2/125 (1.6)	19/361 (5.3)
**Confusion/lack of concentration**	4/100 (4.0)	11/147 (7.5)	2/125 (1.6)	11/361 (3.0)
**Problems speaking or communicating**	0/100 (0)	0/147 (0)	0/125 (0)	6/361 (1.7)
**Headache**	8/100 (8.0)	15/147 (10.2)	10/125 (8.0)	32/361 (8.9)
**Problems seeing/blurred vision**	4/100 (4.0)	2/147 (1.4)	1/125 (0.8)	4/361 (1.1)
**Problems with balance**	2/100 (2.0)	0/147 (0)	1/125 (0.8)	3/361 (0.8)

© 2023 Dun-Dery F et al. *JAMA Network Open.*

**Tremor/shakiness**	1/100 (1.0)	1/147 (0.7)	1/125 (0.8)	3/361 (0.8)
**Cannot fully move or control movement**	1/100 (1.0)	1/147 (0.7)	1/125 (0.8)	1/361 (0.3)
**Tingling feeling/"pins and needles"**	0/100 (0)	1/147 (0.7)	0/125 (0)	2/361 (0.6)
**Miscellaneous**				
**Urination problems**	0/100 (0)	3/147 (2.0)	1/125 (0.8)	4/361 (1.1)
**Bilateral (both eyes) conjunctivitis**	1/100 (1.0)	1/147 (0.7)	0/125 (0)	2/361 (0.6)
**Changes in menstruation**	4/23 (17.4)	6/41 (14.6)	2/51(3.9)	15/122 (12.3)

© 2023 Dun-Dery F et al. *JAMA Network Open.*
