# Supplementary material for: Post–COVID-19 Condition in Children 6 and 12 Months After Infection
Source: JAMA Netw Open. 2023 Dec 28;6(12):e2349613. doi: 10.1001/jamanetworkopen.2023.49613 (PMC10755606; doi:10.1001/jamanetworkopen.2023.49613)
Supplement: Supplement 2. — Nonauthor Collaborators [file jamanetwopen-e2349613-s002.pdf]

Supplemental Online Content: Nonauthor Collaborators
*First name, last name, and suffix (if applicable) are required and will appear in PubMed.

*Group Name(s): Pediatric Emergency Research Canada (PERC) COVID Study Group							
***First Name and Middle Initial(s)**	***Last Name**	***Suffix (eg, Jr, III)**	Academic Degrees	Institution	Location (city, state/province, country)	Role or Contribution, eg, chair, principal investigator	Group (if more than 1 Group listed in the byline) and/or Subgroup (eg, Steering Committee)
Bruce	Wright		MD	University of Alberta	Edmonton, Alberta, Canada	Site Investigator	
Tyrus	Crawford		B.Soc.Sci	Children's Hospital of Eastern Ontario	Ottawa, Ontario, Canada	Database Manager	
Waleed	Alqurashi		MD	Children's Hospital of Eastern Ontario	Ottawa, Ontario, Canada	Scientific Collaborator	
Samina	Ali		MDCM	University of Alberta	Edmonton, Alberta, Canada	Vice-Chair of Research network	
