# Supplementary material for: Post–COVID-19 Condition in Children 6 and 12 Months After Infection
Source: JAMA Netw Open. 2023 Dec 28;6(12):e2349613. doi: 10.1001/jamanetworkopen.2023.49613 (PMC10755606; doi:10.1001/jamanetworkopen.2023.49613)
Supplement: Supplement 3. — Data Sharing Statement [file jamanetwopen-e2349613-s003.pdf]

Data Sharing Statement

Dun-Dery. Post–COVID-19 Condition in Children 6 and 12 Months After Infection. *JAMA Netw Open*. Published December 28, 2023. doi:10.1001/jamanetworkopen.2023.49613

Data
Data available: Yes
Data types: Deidentified participant data
How to access data: Data will be shared, upon reasonable request, for academic purposes, with appropriate individuals who have obtained appropriate ethics permissions and data sharing agreements.
When available: With publication

Supporting Documents
Document types: Statistical/analytic code, Informed consent form
How to access documents: Data will be shared, upon reasonable request, for academic purposes, with appropriate individuals who have obtained appropriate ethics permissions and data sharing agreements.
When available: With publication

Additional Information
Who can access the data: Data will be shared, upon reasonable request, for academic purposes, with appropriate individuals who have obtained appropriate ethics permissions and data sharing agreements.
Types of analyses: Data will be shared, upon reasonable request, for academic purposes, with appropriate individuals who have obtained appropriate ethics permissions and data sharing agreements.
Mechanisms of data availability: Data will be shared, upon reasonable request, for academic purposes, with appropriate individuals who have obtained appropriate ethics permissions and data sharing agreements.
